# Supplementary material for: Challenges and limits of mechanical stability in 3D direct laser writing
Source: Nat Commun. 2022 Apr 19;13:2115. doi: 10.1038/s41467-022-29749-9 (PMC9018765; doi:10.1038/s41467-022-29749-9)
Supplement: Supplementary file 2 — Description of Additional Supplementary Files [file 41467_2022_29749_MOESM2_ESM.pdf]

Description of Additional Supplementary Files

File Name: Supplementary Movie 1
Description: Fabrication of a pyramid polymer network in a cubic monomer pool. Free monomers are shown with a pink glassy material.

File Name: Supplementary Movie 2
Description: Rod printing on a substrate
